# Innovative minimally invasive implants for osteoporosis vertebral compression fractures

**DOI:** 10.3389/fmed.2023.1161174

**Published:** 2023-03-20

**Authors:** Yi Luo, Da-Mei Yang, Hong-Mei Yang, Di Wu, Feng-Ying Xie

**Affiliations:** Department of Pain Medicine, Bishan Hospital of Chongqing Medical University, Chongqing, China

**Keywords:** osteoporosis vertebral compression fractures, kyphoplasty, bone cement leakage, minimally invasive surgery (MIS), pain

## Abstract

With increasing population aging, osteoporosis vertebral compression fractures (OVCFs), resulting in severe back pain and functional impairment, have become progressively common. Percutaneous vertebroplasty (PVP) and percutaneous kyphoplasty (PKP) as minimally invasive procedures have revolutionized OVCFs treatment. However, PVP- and PKP-related complications, such as symptomatic cement leakage and adjacent vertebral fractures, continue to plague physicians. Consequently, progressively more implants for OVCFs have been developed recently to overcome the shortcomings of traditional procedures. Therefore, we conducted a literature review on several new implants for OVCFs, including StaXx FX, Vertebral Body Stenting, Vesselplasty, Sky Bone Expander, Kiva, Spine Jack, Osseofix, Optimesh, Jack, and V-strut. Additionally, this review highlights the individualized applications of these implants for OVCFs. Nevertheless, current clinical studies on these innovative implants remain limited. Future prospective, randomized, and controlled studies are needed to elucidate the effectiveness and indications of these new implants for OVCFs.

## Introduction

With the progression of an aging population worldwide, the incidence of osteoporosis is constantly increasing (1). The number of patients with osteoporosis vertebral compression fractures (OVCFs) is concurrently increasing, with the main symptoms being lower back pain and spinal deformity (2). In additon, OVCFs patients are predisposed to multiple comorbidities, including pulmonary dysfunction, bladder contraction disorder, weight loss, anxiety and depression (3, 4). Furthermore, neurological impairments is also detected in OVCFs patients. Although severe spinal cord injuries are rare in patients with OVCFs, more cases with neurological impairments is conus medullaris damage ascribing to the loss of vertebral body height (5). Owing to low bone mass and devastating of microarchitecture, OVCFs is mainly caused by low energy injury, and a small portion of OVCFs are attributed to high-energy injuries like traffic accident (6).

Currently, OVCFs are treated conservatively or surgically. Conservative treatment involves being bedridden long-term and potential complications such as chronic lower back pain, multisystem infection, and cardiopulmonary dysfunction, leading to an increased mortality rate in elderly patients and affecting quality of life (7). Percutaneous vertebroplasty (PVP) is a minimally invasive procedure for strengthening the vertebrae by injecting bone cement or artificial bone into the vertebra. It was first to treat vertebral angiomas satisfactorily. Subsequently, it was used for gradual OVCFs treatment (8). Although PVP provided good lower back pain relief, the height of the compressed vertebra was not improved by polymethylmethacrylate cement (PMMA) injection alone and the rate of cement leakage was still high (9). Subsequently, percutaneous kyphoplasty (PKP) was developed and found to significantly restore vertebral height, relieve pain, and reduce the incidence of cement leakage (10). The most common PKP procedure is balloon kyphoplasty (BKP), which uses an expandable balloon and PMMA for vertebral restoration and augmentation. However, BKP has some limitations including balloon puncture risk, vertebral body restoration, re-collapse of the vertebral body after balloon withdrawal, and severe cement leakage (11). To solve these problems, many new minimally invasive implants have been designed. This review introduces such innovative implants and highlights their individualized application.

## StaXx FX

When treating OVCFs, good endplate reduction is important for restoring load transfer and reducing adjacent vertebral fracture risk. The StaXx FX system consists of several PEEK wafers (1-mm thick, 8 mm-wide and 30-mm-long) which are implanted into the vertebra through a transpedicular approach. The PEEK wafers overlap with each other in the vertebra controlling the reduction of the compressed vertebra, whereafter PMMA is delivered for stability. Biomechanical studies have shown that the StaXx FX System effectively reduces OVCFs associated kyphotic and endplate deformities. Moreover, StaXx FX restores disk load sharing and, theoretically, reduces the incidence of adjacent vertebral fractures (12).

However, van der Plaat et al. (13) reported the case of a 76-year-old female with a compressed thoracic vertebral fracture treated with the StaXx FX system. After 1 week, the PEEK wafer protruded from the vertebra and almost reached the right pulmonary artery. The patient recovered after displaced wafer removal *via* thoracotomy. This case demonstrates that the StaXx FX System needs further development before being considered safe for OVCFs treatment.

## Vertebral body stenting (VBS)

**FIGURE 1:**
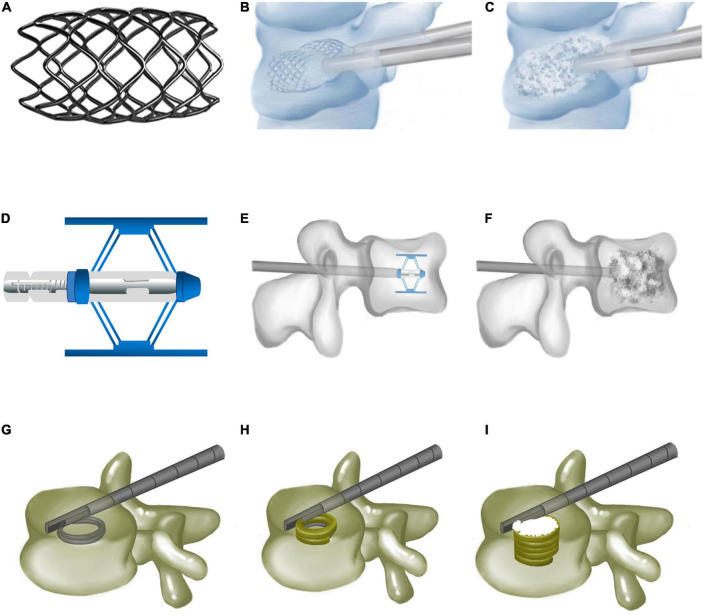
Vertebral body stenting, Spine Jack, and Kiva system. **(A)** Vertebral body stenting appearance. **(B)** Vertebral restoration using vertebral body stenting. **(C)** Bone cements injected after vertebral body stenting expansion. **(D)** Expanded Spine Jack. **(E)** Spine Jack expanded inside the vertebra *via* a transpedicular approach. **(F)** Bone cements augmenting the restored vertebra after the Spine Jack is placed inside the vertebra. **(G)** Guided coil of the Kiva system inserted *via* transpedicular cannula. **(H)** PEEK implant of the Kiva system along the guide wire forming a hollow cylinder inside the vertebra. **(I)** Injected polymethylmethacrylate cement, surrounded by the Kiva system.

The vertebral body stenting (VBS) system consists of a balloon and titanium stent, simultaneously delivered into the vertebra (14) to restore vertebral height through a transpedicular approach. After balloon retraction, the stent remains inside to maintain vertebral height (Figures 1A–C). A comparison of the biomechanical performance of VBS with that of BKP in a vertebral fracture model showed no statistical difference between them regarding mechanical properties. However, VBS better prevented vertebral height loss compared to BKP (15).

Klezl et al. (16) reported the results of 17 patients (7 with OVCFs, 10 with traumatic vertebral fractures) treated with the VBS. The visual analogue scale (VAS) score in the OVCFs group decreased from 8.9 to 2.5 and from 9.7 to 1.6 in the OVCF and traumatic vertebral fracture groups, respectively. Kyphotic deformity was corrected in both groups. Hartmann et al. (17) evaluated the results of 18 patients with incomplete thoracolumbar burst fractures who received VBS. VBS successfully corrected kyphotic deformity and improved VAS, Oswestry Disability Index (ODI), and SF-36 scores. Comparing the effects of VBS and PVP in OVCFs, Thaler et al. (18) concluded that VBS provides better vertebral height restoration than PVP, with a lower cement leakage rate (1.36 vs. 11.5%). Capel et al. (19) found that the incidence of cement leakage with BKP was the same (50%), with no cases producing associated symptoms. However, the amount of cement leakage was significantly lower with VBS than with BKP. A randomized controlled trial (RCT) comparing VBS and BKP for OVCFs concluded that these did not differ significantly regarding kyphotic angulation correction, cement leakage rate, radiation exposure time, and neurological complications, although the intraoperative balloon expansion pressure was significantly higher in the VBS than the BKP group (20). Furthermore, the VBS system had more material-related complications. Accordingly, they concluded that VBS cannot provide a significant advantage over BKP.

In the upper thoracic and lower cervical spine, bilateral VBS implantation is not recommended because of the small size of the vertebral body. However, CT-guided unilateral VBS implantation is used to reduce operative time and pedicle damage (21). Although PMMA can provide strong initial stability, its disadvantages include high temperature, cytotoxicity, and non-resorbability. Moreover, its stiffness leads to adjacent secondary vertebral fractures. To avoid these problems, calcium phosphate has been used instead of PMMA for VBS and BKP, though without significant differences regarding VAS, ODI, and PMMA cement leakage improvements. However, VBS was more advantageous in vertebral height restoration (22). Detailed preoperative planning should be performed to accurately place the VBS system centrally in the vertebra.

## Vesselplasty

Vesselplasty is an effective alternative to BKP, composed of a special polyethylene terephthalate container (Vessel-X) instead of a balloon. For the procedure, Vessel-X is inserted into the vertebra *via* a transpedicular approach and expanded by PMMA injection to restore the height of the compressed vertebra. Theoretically, because most of the PMMA is contained in the Vessel-X, Vesselplasty avoids bone cement leakage.

Flors et al. (23) used Vesselplasty to treat 29 patients with OVCFs, tumor bone metastases, myeloma, and high-energy trauma fractures, with postoperative VAS score reduction from 8.72 to 3.38, pain and mobility improvements, and no symptomatic complications. He also found Vesselplasty to provide limited vertebral height restoration. Therefore, it is recommended for mild OVCFs. Chen et al. (24) followed up 62 patients who underwent Vesselplasty showing that it improved VAS and ODI scores, restored vertebral body height (from 15.64 to 22.15 mm), and significantly corrected the Cobb angle. Comparing Vesselplasty with PVP for OVCFs treatment, the cement leakage rate was significantly lower with Vesselplasty than with PVP (29.4 vs. 67.4%) (25). Although Vesselplasty can reduce cement leakage, serious cement leakage has been reported. A 77-year-old female underwent bilateral Vesselplasty for T6 OVCF, resulting in large PMMA leakage into the spinal canal and requiring reoperation (26). The Vessel-X rupture was believed to be closely related to heat production during PMMA polymerization. Since bone cement temperature monitoring is difficult after cement injection, the PMMA temperature should be monitored.

## Spine Jack

A Spine Jack is a retractable titanium expander used to restore compressed vertebrae. The unexpanded Spine Jack is cylindrical (5-mm-thick, 25-mm-long) to facilitate vertebral implantation (Figures 1D–F). Once a Spine Jack is implanted, its upper and lower metal plates are simultaneously expanded to a maximum height of 17 mm. Depending on its design, it may expand the upper and lower endplates parallelly to complete vertebral height restoration. The Spine Jack is delivered into the compressed vertebra *via* a transpedicular approach, then expanded to achieve vertebral restoration, and left inside with bone cement, or autologous. Biomechanical tests revealed that Spine Jack restored 96 and 101.3% of anterior and central vertebral body heights, respectively, compared to 85.56 and 93.89% with BKP (27). Under cyclic loading, the Spine Jack maintained 98.56% of vertebral height vs. only 92% with BKP, proving its superiority in restoring vertebral height.

A case series looked at the one-year clinical outcomes of Spine Jack for OVCFs in 103 patients. A total of 81.5% back pain relief, 91.3% ODI score reduction, and 2.9% incidence of adjacent vertebral fractures were observed (28). Similar results have been reported in other clinical studies (29, 30). Lofrese et al. (31) applied Spine Jacks to A2–4 thoracolumbar fractures finding that patients older than 60 years may experience poorer kyphotic deformation correction and a higher surgery-related complications rate. Furthermore, patients who undergo surgery within 7 days post-injury have better clinical outcomes. In an extremely unstable A3 vertebral fracture, the Gardner angle could be corrected while the anterior, central, and posterior vertebral heights could be restored using a Spine Jack plus posterior screw-rod system (32). Spine Jack can also be used for tumor-induced VCFs. In 13 patients with tumor-induced VCFs treated with Spine Jacks, vertebral body height was restored up to 5.6 mm (33). Similarly, the VAS score decreased from 5.5 to 1.5, with six cases of bone cement leakage and one adjacent vertebral fracture (occurred at the 6-month follow-up).

The efficacy of a Spine Jack and PVP for OVCFs was compared in 74 patients (34). Both methods significantly relieved pain without significant differences between the two. However, the Spine Jack was significantly better than PVP in improving kyphotic and Cobb angles. A RCT evaluated the effect of Spine Jack and BKP on OVCFs (35). After a 3-year follow-up, the Spine Jack had a higher EQ-5D score (0.93 vs. 0.81) and was superior to PKP in improving vertebral height and kyphosis. Biomechanical tests and clinical studies have confirmed the Spine Jack system’s effectiveness in treating OVCFs, particularly in restoring and maintaining vertebral height and stability.

## Kiva

The Kiva system was designed to prevent bone cement leakage by surrounding bone cement (36). It is composed of a Nitinol guidewire and a spiral PEEK implant to block bone cement. The guidewire is delivered into the vertebra through a transpedicular cannula and prefabricates a ∼15-mm-thick spiral coil. The PEEK implant along the guidewire forms a 20-mm-thick hollow cylinder. The implant may be accurately adjusted to restore vertebral height. Finally, PMMA is injected to augment the vertebra after guidewire removal (Figures 1G–I).

Clinical results of 26 patients with OVCF treated with Kiva showed that back pain scores decreased from 8.0 to 3.0, while ODI scores improved by 56% (36). PMMA leakage complications were observed in two cases. Another study that observed 57 patients with OVCFs treated with Kiva also found it to be safe and effective (37).

A RCT comparing Kiva and BKP in 190 patients with OVCFs demonstrated that Kiva improved vertebral height and wedge angle. The clinical efficacy of Kiva and BKP was not significantly different. But the leakage rate was lower in the Kiva group, though not significantly. Similar results were obtained in other RCTs (38, 39). Nevertheless, Kiva had shorter operative time and lower risk of adjacent vertebral fractures. Another advantage of Kiva over BKP is its reduced medical cost. A cost consumption comparative analysis between Kiva and BKP including 304 patients found the incidence of postoperative adjacent vertebral fractures to be significantly lower with Kiva. Therefore, the total cost was lower with Kiva than with BKP over the two-year follow-up period (40).

## Osseofix

**FIGURE 2:**
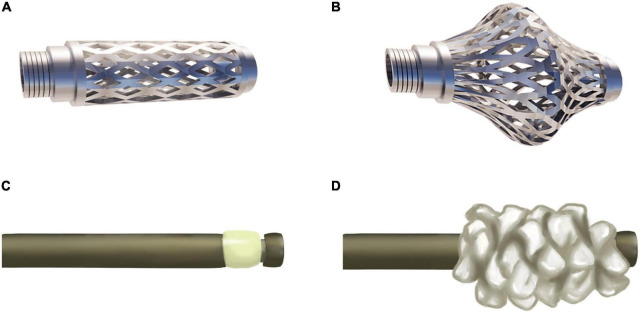
Osseofix and Sky Bone Expander. **(A)** Unexpanded Osseofix. **(B)** Expanded Osseofix. **(C)** Unexpanded Sky Bone Expander. **(D)** Expanded plastic polymer of the Sky Bone Expander.

As an intravertebral titanium mesh implant, the Osseofix system can restore compressed vertebrae and provide mechanical strength for stability (Figures 2A, B). Osseofix is inserted into the vertebra *via* a transpedicular or extra-pedicular approach. Subsequently, the titanium meshes are expanded to restore vertebral height. Finally, PMMA is injected for stability. Although biomechanical experiments revealed the same biomechanical performance for Osseofix and BKP (41), Osseofix maintained better vertebral height using significantly less PMMA.

Ender et al. (42) observed 32 patients with OVCFs treated with Osseofix. At the 1-year follow-up, ODI scores decreased from 79.6 to 30.1%, the vertebral body kyphotic angle improved from 11.7 to 10.3°, and only one patient experiences postoperative vertebral height loss. No adjacent vertebral fractures occurred. A study of 16 patients with multiple myeloma undergoing surgery with Osseofix elicited VAS score decreases from 8.6 to 3, and ODI score improvements by 34.1 points (43). No complications occurred, including bone cement leakage. It was ascribed to Osseofix requiring less bone cement.

To eliminate cement leakage, researchers have attempted to use Osseofix without PMMA for OVCFs. A study comparing the biomechanical performance of the BKP, and Osseofix with and without bone cement showed no significant differences among them (44). However, notably, *in vitro* mechanical tests provide little information on clinical symptom relief. To address this, Eschler et al. (45) reported four cases of OVCFs treated with Osseofix without bone cement. At the 1-year follow-up, VAS score decreased from 8.8 to 2, kyphotic angle improved from 14.5 to 10.7°, and Cobb angle decreased from 10.7 to 8.3°. Although a small loss of reduction was observed, Osseofix without bone cement was deemed effective for OVCFs.

## Sky bone expander (SBE)

Controlling the intravertebral cavity shape created by the balloon is difficult in the BKP procedure. Thus, precise compressed vertebra restoration is challenging. Furthermore, intractability issues due to an overly inflated balloon, leading to vertebral reduction rupture are common. The SBE system was developed to overcome these drawbacks. In this system, the plastic polymer expander, is adopted to restore vertebral height and create a cavity for PMMA injection (Figures 2C, D). The advantages include precise control of the area and restoration height. Moreover, SBE restores the vertebra along the longitudinal axis, contributing to reduced vertebral lateral wall damage.

Tong et al. (46) reported 9 patients with thoracolumbar fractures who experienced significant pain relief after SBE. Foo et al. (47) observed 40 patients with vertebral fractures treated with SBE. Vertebral height was restored, the kyphotic angle improved, and pain significantly relieved. Asymptomatic cement leakage occurred in three patients and one adverse event of difficult device removal occurred. Thus, the dilator tube must be maintained in the proper position (3 mm from the anterior wall and 5 mm from the lateral wall). Most importantly, when adjusting the dilator tube, the SBE must be completely contracted before moving it.

## Optimesh

The Optimesh system is a retractable mesh capsule filled with allogeneic and autologous bone instead of bone cement (48). The Optimesh system was found to be able to restore vertebral height and provide mechanical stability (49). Moreover, allogeneic or autologous bone, unlike bone cement achieves modulus elasticity similar to that of normal vertebra, and may reduce adjacent vertebral fracture risk, especially in younger patients. Inamasu et al. (50) reported one patient with vertebral fracture treated with Optimesh plus screw-rod. The patient achieved 3.0 and 3.5 mm of anterior and central vertebral height restoration, respectively, with quickly improved back pain. The patient returned to normal work 3 months postoperatively, without residual lower back pain at the 1-year follow-up. Currently, there is still little clinical evidence for the efficacy of the Optimesh system.

## Jack

The Jack system incorporated a cephalic metal expander and posterior control handle, allowing parallel expansion of the upper and lower endplates. Once Jack is placed in the compressed vertebra *via* a transpedicular approach, bone cement is injected for augmentation.

Mechanical testing has shown no significant statistical differences between Jack and BKP regarding biomechanical parameters restoration (51). Both systems may restore vertebral body height, but Jack uses less PMMA. In a study involving 218 patients with OVCFs treated with Jack, at a mean follow-up of 14.2 months, VAS scores decreased from 8.2 to 1.8, ODI scores decreased from 78.2 to 20.9%, and central vertebral height recovered from 18.7 to 24.5 mm (52). Another clinical study compared Jack with BKP for OVCFs (53). The Jack system was significantly better than BKP in improving anterior vertebral height, central vertebral body height, and Cobb angle.

## V-strut

The new V-strut system is used for the treatment of vertebral fractures caused by osteoporosis or tumors. It is a cylindrical implant made of PEEK material with a small hole at the front for anchoring into the vertebral body. Once V-strut is implanted, PMMA is injected into the vertebra for augmentation.

V-strut advantages include rapid pain relief, improved function, and vertebral height maintenance. Although V-strut cannot restore vertebral height, it can maintain upper and lower endplate positions, resist axial stress, and prevent vertebral refracture. The V-strut system exhibits the same biomechanical properties as PVP, both meeting the requirements for OVCFs (54). Cornelis et al. reported six patients with vertebral fracture (five tumors, one trauma) treated with V-strut. VAS and ODI scores decreased from 6.2 to 1.7, and 36 to 23, respectively. Four cases of asymptomatic cement leakage occurred (55). The therapeutic efficacy of V-strut still requires further observation. However, this system is a highly promising, minimally invasive implant for OVCFs and cancer-related fracture treatment (56).

## Conclusion and perspectives

In the last two decades, new implants for OVCFs treatment have been introduced, to improve upon PVP and PKP. Each of these innovative implants has unique characteristics and indications. The Jack, SBE, and Spine Jack achieve remarkable vertebral height restoration. VBS and Osseofix maintain vertebral height after restoration. Kiva and StaXx FX precisely strut the compressed vertebra. Vesselplasty and Optimesh effectively reduce PMMA leakage incidence. The V-strut system is recommended for osteolytic fractures caused by vertebral tumors. Personalized applications should consider the advantages and disadvantages of these implants. However, some problems must still be addressed, such as the risk of neurological damage caused by the surgical technique, implant biocompatibility, and excessive difference in mechanical properties between the implant and vertebral body. Therefore, newly designed implants need to follow these principles: (1) ensure appropriate mechanical strength to maintain vertebral stability without causing secondary fractures in the adjacent vertebrae; (2) have good biocompatibility. Moreover, clinicians must explore novel surgical techniques and approaches to reduce the occurrence of puncture complications. We believe that as technology and biomaterials continue to improve, minimally invasive surgery for OVCFs will become safer and more effective.

## Author contributions

All authors listed have made a substantial, direct, and intellectual contribution to the work, and approved it for publication.
